# Mapping Healthcare Needs: A Systematic Review of Population Stratification Tools

**DOI:** 10.3390/medsci13030145

**Published:** 2025-08-19

**Authors:** Giovanni Genovese, Caterina Elisabetta Rizzo, Antonio Nirta, Linda Bartucciotto, Roberto Venuto, Francesco Fedele, Raffaele Squeri, Cristina Genovese

**Affiliations:** 1 Department of Biomedical Sciences and Morphological and Functional Images (BIOMORF), University of Messina, Via Consolare Valeria, 1, 98124 Messina, Italy; gigenovese@unime.it (G.G.); antonio.n2396@gmail.com (A.N.); lindabartucciotto@gmail.com (L.B.); f.fedele1965@libero.it (F.F.); squeri@unime.it (R.S.); 2 Department of Prevention, Provincial Health Agency of Messina, 98123 Messina, Italy; roberto.venuto@hotmail.it; 3 National Center for Clinical Governance, National Health Institute, 00161 Rome, Italy; 4 Department of Chemical, Biological, Pharmaceutical and Environmental Sciences (CHIBIOFARAM), University of Messina, 98166 Messina, Italy

**Keywords:** population health management, stratification, clinical risk groups, advanced clinical risk group, health management

## Abstract

**Background and Aim**: In 2021, healthcare expenditure in Italy represented 7.3% of the national gross domestic product, with approximately 80% attributed to the management of chronic diseases—an increasing burden associated with population aging. Population stratification tools have emerged as critical instruments for the efficient allocation of healthcare resources, particularly for high-need, high-cost individuals. This systematic review aimed to identify, classify, and evaluate existing population stratification tools based on their characteristics, validation status, and practical applications. **Methods**: A systematic literature review was conducted in accordance with the PRISMA 2020 guidelines to examine adult population stratification models grounded in healthcare needs. The review encompassed studies retrieved from major scientific databases and included both national and international implementations. **Results**: The initial search yielded 140,111 records, from which 17 distinct stratification tools were identified. Of these, nine had undergone validation through peer-reviewed studies. Within the Italian context, only six tools were in active use—three of which were developed as region-specific algorithms, while the remaining three employed internationally established software platforms. **Conclusions**: Population stratification tools provide a robust framework for assessing both clinical complexity and resource utilization, thereby facilitating the design of integrated care pathways and evidence-based policy decisions. In the context of proactive and personalized healthcare delivery, such tools play a pivotal role in enhancing system efficiency, informing strategic planning, and promoting equitable access to care.

## 1. Introduction

In 2021, national healthcare spending accounted for 7.3% of Italy’s gross domestic product, amounting to approximately EUR 127 billion [1], with 80% attributed to chronic diseases [2]. Among the variables that currently exert the most significant influence on the management of population health, there are the clinical complexity of patients, the aging population and the consequent sustainability of our universal healthcare system [3]. While some individuals can be classified as healthy, others experience significantly compromised health. The former group incurs minimal or no costs to the healthcare system, whereas the latter group consumes a substantial amount of resources [4]. Additionally, it is important to note that even among individuals suffering from the same pathology, there will not be an exact correspondence in their health conditions. This situation is exacerbated by the aging population and the increasing demand for assistance [5]. Distinguishing between these groups is a significant challenge for planning that is attentive to care while being compatible with the resource expectations and health needs of patients [2].

Given the difficulty in securing new economic resources, efficient management of existing resources becomes essential while simultaneously ensuring the provision of care that meets the patient’s needs [6]. Population stratification methods based on care complexity constitute a valuable tool for the appropriate planning of services, particularly for those individuals with higher resource consumption. Considering clinical complexity requires identifying patients with one or more chronic diseases, which is now possible thanks to current administrative information systems [7]. It is important to recognize that individuals affected by the same disease exhibit varying levels of severity and therefore require different treatment pathways. Population health management (PHM) is a people-centered, data-driven, and proactive approach to managing the health and well-being of a defined population, considering the differences within that population and the social determinants of health. Population health management entails a data-driven assessment of a specific population’s health status, followed by predicting the health outcomes and anticipating the resources needed to address those outcomes proactively [8].

However, initiatives at both the national and global levels remain limited, likely due to policymakers’ lack of awareness of population stratification tools and, in some cases, their high cost [9].

In Italy, the issue is exacerbated by the aging population, the ongoing economic crisis, and the high toll of the COVID-19 pandemic. Consequently, we were motivated to conduct a systematic review to identify all the available stratification tools, classifying them based on their characteristics and uses, not only at the national level but also globally.

## 2. Materials and Methods

A systematic review of the literature was conducted following the PRISMA 2020 [4] guidelines, aiming at evaluating the use of adult population stratification tools based on health needs at a national and global level. The search was conducted in the scientific databases PubMed, Cochrane, and Web of Science for every article published until June 2025. These databases were chosen for their comprehensive coverage of the biomedical, clinical, and health sciences literature, ensuring a thorough and robust search for a systematic review on this topic. The PRISMA 2020 Main Checklist and the PRISMA Abstract Checklist are included as Supplementary Materials to this review [4].

The acronym PICO (patient, intervention, control, and outcome), whose application is exemplified in Table 1, effectively summarizes the phases of the process.

The search strategy involved finding, at an international scale, all the studies, published or not, that concerned the topic. The searches were conducted considering many different sources: electronic databases (Medline, Embase, Cinahl, etc.), national and international registers, non-indexed periodicals, and bibliographic references of the included studies. Once the eligible studies were identified, the inclusion criteria were applied to the full texts. The study’s inclusion criteria evaluated the type of patients, interventions, comparisons, and outcomes, in addition to the study design. The clinical heterogeneity was examined to derive a pooling of results in the meta-analysis. Finally, the type of statistical heterogeneity, which concerned significant differences in the results of the studies, was evaluated using the Higgins test. The effect of these choices on the results was subsequently evaluated via a sensitivity analysis, which verified the consistency of the results when the inclusion parameters varied and described them as robust (non-variable) or sensitive (variable) with respect to each parameter.

All the identified tools were characterized in terms of the population stratification methodology, data segmentation method, presence of peer-reviewed validation, need for computerized electronic health records, and proprietary status of the devices under investigation, as shown in Table 2.

### 2.1. Inclusion and Exclusion Criteria

“Healthcare needs-based model” was defined as any systematic tool or algorithm that segments a population based on factors such as clinical diagnosis, disease complexity, functional status, or historical healthcare resource utilization. The primary purpose of such a tool must be to predict or stratify individuals according to their current or future need for healthcare services.

All the investigated studies were evaluated based on the following inclusion/exclusion criteria.

A. Inclusion criteria

Study on PHM methods that investigates the role of the tool in the stratification of a population adopted at the national or local level.

B. Exclusion criteria
  1. Duplicate studies
  2. Systematic reviews

### 2.2. Selection Process

We decided to include studies based on PHM at national or local levels. All the authors screened each record and each report retrieved; each author worked independently on this task. Giovanni Genovese (G.G.) and Caterina Elisabetta Rizzo (C.E.R.) independently conducted the screening of the titles and abstracts, as well as the revision of the complete texts. Any disagreements were resolved through discussion with a third author (Antonio Nirta—A.N.) until consensus was reached.

## 3. Results

**Figure 1:**
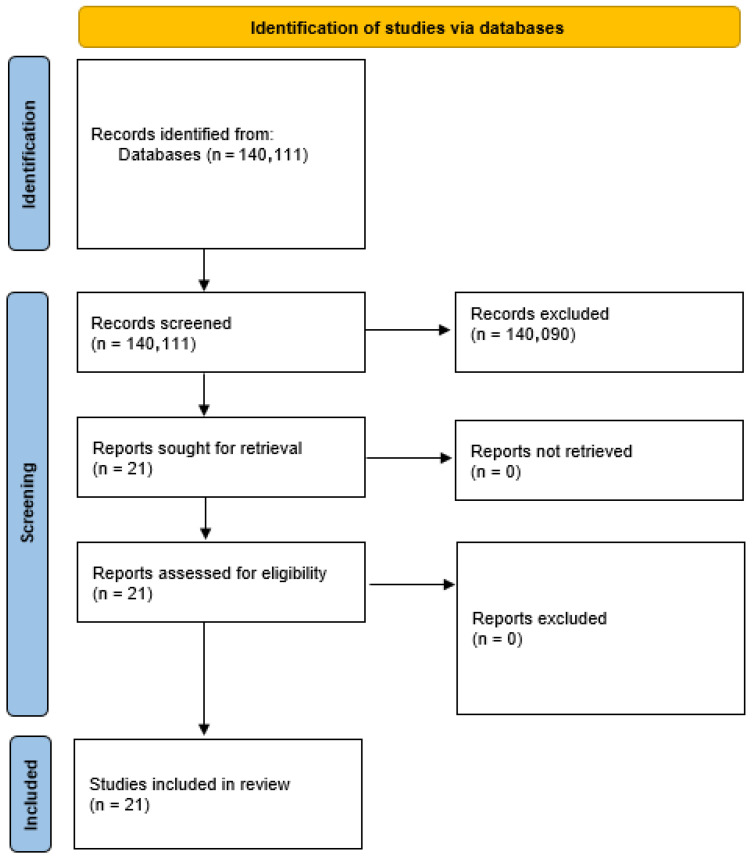
Flowchart of the evaluation and inclusion process for the systematic review.

A total of 140,111 articles were identified and analyzed to assess whether the objectives of this study were consistent with what we analyzed, and after the removal of duplicate and irrelevant articles, 21 were subjected to review (Figure 1).

Search term sample inputs for PubMed:

((community* OR population*)

AND

(“health need*” OR “health service* need*” OR “healthcare need*” OR “healthservice* need*” OR “health care need*” OR “health care service* need*” OR “medical care need*” OR “medical service* need*” OR “biopsychosocial need*” OR “bio-psychosocial need*” OR “health requirement*” OR “healthservice* requirement*” OR “healthcare requirement*” OR “healthcare service* requirement*” OR “health care requirement*” OR “health care service* requirement*” OR “medical care requirement*” OR “medical service* requirement*” OR “biopsychosocial requirement*” OR “bio-psychosocial requirement*” OR “healthcare utilisation” OR “health service utilisation” OR “healthcare service utilisation” OR “health care utilisation” OR “health care service utilisation” OR “medical care utilisation” OR “medical service utilisation” OR “healthcare utilization” OR “health service utilization” OR “healthcare service utilization” OR “health care utilization” OR health care service utilization” OR “medical care utilization” OR “medical service utilization” OR “health risk*” OR “health status*” OR “health profile*” OR “biopsychosocial risk*” OR “biopsychosocial status*” OR “biopsychosocial profile*” OR “bio-psychosocial risk*” OR “bio-psychosocial status*” OR “bio-psychosocial profile*”)

AND

(typolog* OR stratification* OR segmentation* OR classification* OR categorization* OR categorisation*))

Peer-reviewed validation studies were found for nine of the algorithms, while only six applications were used on the national territory. Three of these six applications have seen the development of ad hoc algorithms at a regional level, while the other three used software already existent at an international level.

The first model proposed by Lynn et al. divided the population into eight groups: people in good health, maternal/infant situations, with an acute illness, with stable chronic conditions, with a serious but stable disability, with failing health near death, with advanced organ system failure, and with long-term frailty. Since this framework would guide each population segment across the institute’s “Quality Chasm”, it is called the “Bridges to Health” model [5].

The second one, proposed by Hewner et al., was able to demonstrate a reduction in the hospitalization rates due to risk-stratified care management and the number of layers was four. This approach demonstrated an effective way to translate evidence-based research to the Medicare population, smoothing the transition back into the community and preventing avoidable hospital admissions [24].

In the Kaiser Permanente model, the need for healthcare assistance was classified into three levels [25].

The model of Joynt et al. divided the population into six groups at high or low risk. High-cost patients were more often female, less often white, much more often dually eligible, and slightly more likely to live in urban areas. They were also much more likely to have a mental health or substance abuse diagnosis and to have a higher median number of chronic medical conditions. The high-cost patients in each group were more likely to have higher rates of mental health and chronic medical conditions, though the absolute rates of these elements differed across segments [13].

The Delaware population clustering used electronic data and was an unvalidated model, led by experts, and the population was stratified into 20 segments [10].

The Britannic model was based on socio-demographic conditions, chronic diseases, and summaries of healthcare services each resident used each year. After this, the population was divided into 14 health status categories ranging from Healthy to End-Of-Life according to their highest healthcare needs each year [10].

The Singapore model distinguished people with the following conditions: “Mostly healthy”, “Serious acute illness but curable” and “End of life” [which are also present in the Ministry of Health Singapore framework]. Patients with chronic diseases were segmented into “Stable chronic disease”, “Complex chronic diseases without frequent hospital admissions” and “Complex chronic diseases with frequent hospital admissions” [14].

The Model of North West London stratified the population into 10 segments and included functional ability too [26].

Van der Leen et al. used the factor mixture model to categorize the respondents into segments with relatively similar experienced difficulties concerning their functioning. First, the analyses showed that older adults can be empirically categorized into five meaningful segments: feeling vital; difficulties with psychosocial coping; physical and mobility complaints; difficulties experienced in multiple domains; and feeling extremely frail. More precisely, the indicators employed were selected from the following two validated instruments: the Groningen Frailty Indicator [GFI] [15,27] and INTERMED [28]. They selected the subscales “biological” [five items] and “psychological” [five items] from the INTERMED to measure a person’s felt needs in the physical and psychological domain of human functioning because these subscales were more extensive compared to the questions from the GFI covering these domains.

The 3M™ Clinical Risk Groups classification methodology describes the health status and burden of illness of individuals in an identified population. The optional 3M Functional Status Groups [FSG] methodology supplements the 3M CRGs when individuals have limitations in performing the activities of daily living [12].

Liu et al. adopted a system based on 16 health status indicators [i.e., diabetes, renal disease, heart disease, stroke, comorbidity, etc.] [16].

La Fortune et al. used a system that divided the population into four main categories based on 17 indicators [17].

In the study conducted by Vuik et al., eight population segments were identified, and the utilization of each care setting was significantly different across all the segments, showing that utilization-based cluster analysis provides a quantitative evidence base to improve population health [18].

The Singapore Health Services Regional Health System was used in the study conducted by Low et al., in which the data utilization included demographics, chronic disease status public healthcare utilizations, specialist outpatient visits, emergency department visits, primary care clinic visits, and mortality [19].

Davis et al. conducted a retrospective observational cohort study, using latent class analysis to identify clusters of alike patients based on 53 hierarchical condition categories [20].

Whitson et al. conducted a study based on an LCA of 14,052 Medicare beneficiaries based on self-reported variables capturing 13 chronic conditions [29].

In the study by Dorr et al., six primary care practices risk-stratified their entire patient population over 2 years. The individual patient risk scores created by the practices were collected and compared to a common risk score in terms of their ability to predict future expenditures, emergency department visits, and hospitalizations [22].

Dumini et al. developed the Cross-Country Simple Segmentation Tool: using clinical indicators, self-reported physician diagnosis of chronic disease, and performance-based tests conducted during the survey interview, individuals were assigned to 1–5 global impression segments and assessed for having any of the four identifiable complicating factors [21].

### 3.1. Models Applied in Italy

#### 3.1.1. Lazio Region

One of the first attempts to apply a stratification system in Italy comes from the Lazio Region [30]. Three population strata were identified through this stratification system: people with more than one chronic pathology, people with multi-chronicity and high clinical complexity, and people with multi-chronicity with socio-economic vulnerability factors. The positive predictive value was extremely low: 46.3%, 16.3%, and 30.3%, respectively, for the three layers analyzed. The study demonstrated that the ability to stratify and predict the health needs of a population through the hospital information flow alone is very limited.

#### 3.1.2. Emilia-Romagna Region

The RiskER project aimed at developing a statistical model based on administrative information flows, which would allow the identification of risk measures [or predictions for the use of services] useful for the stratification of the population concerning the articulated and complex levels of the case mix. Starting from the vast wealth of administrative information flows, the Emilia-Romagna Region has developed a statistical model called RiskER [23] that offers the possibility of defining the degree of clinical and healthcare complexity of the population subject to healthcare activities and identifying different risk bands of frailty of the population, particularly those at high and very high risk.

#### 3.1.3. Lombardy Region

The Lombardy Region has launched a stratification method based on 62 chronic pathologies. The level of complexity indicates the number of pathologies present at the same time, and in particular, the first level corresponds to more than three pathologies; the second level to two or three pathologies; and the third level to mono-pathology [11].

#### 3.1.4. Umbria Region

The Umbria Region and the Lombardy Region have used the 3M CRG system [12]. This system allows the classification of the entire population according to clinical logic based on the skills of experts, using diagnosis and procedure data, pharmacological therapies, and other information, including personal details, present in the current administrative flows.

Thanks to the grouping of data, based on a clinical categorical method, individuals are therefore assigned to homogeneous risk groups based on the severity of the disease, and these groups are mutually exclusive.

There are 1080 groups and this allows a high level of granularity in terms of the patient groups, but it is also possible, if desired, to reduce the number of groups by aggregating them with the same clinical significance and the same criteria for adjusting to the severity of the disease.

The choice of the level of aggregation is linked to the need, for example, to extrapolate information relating to the population affected by multiple comorbidities or chronic conditions.

#### 3.1.5. Veneto Region

The Veneto Region has used the ACG ^®^ system developed by Johns Hopkins University [31]. This tool is a territorial grouper [just as the DRG is a grouper for the hospital], which stratifies the case mix of the population based on the diseases co-present in each person, thus mapping their distribution and impact on the territory in relation to the use of health resources.

The ACG group split the population into 93 ACG categories, postulated by iso resources, which classify all the subjects in a mutually exclusive manner based on the diagnoses and prognostic trends of diseases. The population can then be stratified into six categories, defined as RUBs [resource utilization bands], which identify the six main strata into which a population can be divided.

The VDA Proximity GPI system was adopted by the Valle d’Aosta Local Health Authority within the ADIUVANT project [ADvanced Ict cloUd-based and Virtualized platform for integrated and Personalized Medicine]. The trial involved chronic patients with cardiovascular, neurological, and pneumatological diseases, who were enrolled at the Pavia and Bari centers of the IRCCS Maugeri. The project involved the prototyping, testing, and validation of a technological platform for the governance of chronic conditions, with specific reference to disabling pathologies with a strong social impact: cardiovascular, neurological, and pneumological. The platform was intended to support patients and healthcare workers with IT tools and services dedicated to improving the entire treatment path and monitoring the adherence and effectiveness of treatments during the rehospitalization phase, in line with the clinical pathway.

## 4. Discussion

### 4.1. Summary of Main Results

The algorithms had very many layers, leading to high associated data frequency and higher sensitivity. There have been only two applications tested in European and North American countries [CRG and ACG], both with a minimal risk of bias [12,31]. Other studies reported the use of CRG in Italy, but we find these in the gray literature; in particular, the system, developed by 3M in the second half of the 1990s, was introduced in 2012 by the Health Information System [HIS] division in Italy. It was used, evaluated and validated for the first time as part of the Ministry’s finalized research project “RF-2009-1483329-Utilization of Regional Health Service databases for evaluating epidemiology, short and medium-term outcome, and process indexes in patients hospitalized for heart failure” carried out by the Niguarda Ca’ Granda Hospital on behalf of the Lombardy Region. The results were disseminated via oral communications at two conferences [“Healthcare Databases and Population-based Case-Mix Systems to Support Decision Makers on Heart Failure Management in an Italian Region”, held in July 2015 in the Netherlands, and “Incidence, outcomes, and process of treatment of heart failure in Lombardy. The evidence from health information systems outlines a new plan”, held in 2015 in Milan].

The system was then adopted by the Region of Umbria as a pilot experimental project for the stratification of the resident population of one of the two LHUs based on health status and healthcare needs adjusted according to clinical risk. The results were presented at several conferences [“A new classification system for the patient population: a pilot study in the USL Umbria 2”, May 2016–Foligno Hospital—Terni; “Testing of a health planning tool in the USL Umbria 2: the 3M CRG classification”, October 2016–42nd ANMDO Congress—Bologna; “Management of chronic conditions through the use of the clinical risk group classification system: a pilot study in the USL Umbria 2”, November 2016–49th SITI National Congress—Naples; “The role of the clinical risk group classification system in the management of oncological patients in need of high complexity of care. A pilot study in the local health unit”, October 2017–AIOM National Congress—Rome].

Also, the LHU of Catanzaro, the ASReM of the Molise Region and the LHU Napoli 3 Sud have adopted the CRGs to process the data on their patient population to derive homogeneous risk-adjusted groups to optimize the use of healthcare resources and support healthcare planning in the short and long term through the early identification of subjects potentially at risk, accurate clinical and longitudinal management of the disease, predicting future spending on care and measuring the performance of the various healthcare providers.

### 4.2. Generalizability of the Retrieved Results

The segmentation of the population based on healthcare needs is important to optimize the allocation of economic resources and inform the services available; in this context, policymakers play a fundamental role in the distribution of resources to optimize the health of the population [4].

Healthcare needs can be used to stratify a population design healthcare intervention for patients in low-need segments and evaluate the outcomes based on their progression to higher-need segments of the population [32,33], and this could be important to evaluate social health services and preventive ones, quantifying effectiveness by reducing the probability of progression to higher-morbidity segments [34].

Therefore, this provides a new means to evaluate the ability of the healthcare intervention to meet the health needs [i.e., effectiveness] of specific population segments.

The advantages that have emerged in the application of these methods are various, including their usability with current administrative flows, the classification of all patients allowing tracking of the prevalence and progress of chronic diseases, supporting short- and long-term health planning through the early identification of subjects potentially at risk, analyzing the clinical effectiveness of clinical pathways, optimizing the use of healthcare resources by reallocating them, promptly monitoring planned spending, predicting future care, and finally, measuring the performance of the various providers of healthcare services [35].

At a national and international level, therefore, a more punctual and precise application would be needed in the presence of homogeneity in the classification of diseases with international institutions. In Italy, only 20 years after the introduction of the Diagnosis Related Groups [DRGs], the system for the remuneration of hospital services in the NHS [36] was introduced.

With this aim in mind, the project to develop a new system for measuring and valorizing hospital products [It. DRG Project] [37] was born, which should provide the NHS with a new system for measuring and valorizing hospital products, based on a rigorous and transparent methodology and based on data observed in Italian hospitals.

Centralizing the use and application of stratification systems is crucial; for instance, in monitoring pharmaceutical expenditure.

### 4.3. Implications for the Daily Practice of Potential Readers [Including Public Health Professionals]

At the national level, it is necessary to adopt a unique stratification model [as we have observed, there are a few attempts, mainly in the regions of North and Central Italy]. The National Recovery and Resilience Plan [PNRR]’s strategic objective is to guarantee consistent language usage with guarantees of more equitable and homogeneous access [38].

It is crucial to note that in the application of these models, the “human” stratification was the least sensible and specific, and it had a lower positive predictive value. This necessitates the implementation of standardized systems at the international level to facilitate comparisons.

Finally, as regards stratification systems, their use and application at a central level is important (such as application in monitoring pharmaceutical spending).

Furthermore, a single stratification model should be adopted at a national level: this is one of the strategic objectives of the PNRR, as it would allow for uniformity of the language used, with guarantees of fairer and more homogeneous access.

To conclude, the stratification of the entire population and the assignment of each citizen to a different state of health and subsequently to a homogeneous group of patients with similar care needs is the first step toward a different measurement of care needs [39,40]. The organizational implications that are an integral part of today’s debate on the reorganization of services and population health management are evident. In terms of measuring activities and their outcome indicators, the patient would thus once again become the common denominator.

### 4.4. Limitations

This review, while complete, is subject to several limitations. First, our research was limited to studies published in English and Italian, introducing a potential linguistic bias that may have led to the omission of relevant tools developed and validated in other linguistic contexts. Furthermore, the results may be influenced by a publication bias, since studies reporting neutral or negative results for certain stratification tools are less likely to be published, which may skew the available evidence in favor of more positive outcomes. Finally, although we included some findings from the gray literature, our research in terms of these sources was supplemental and not exhaustive; as a result, it is possible that some non-peer-reviewed tools or important reports from government agencies and healthcare organizations were not included in our analysis.

### 4.5. Validity

The validation methodologies used for the instruments examined mainly fall into two categories:

1. Predictive validity: This was the most common approach, where a tool’s performance was assessed based on its ability to accurately predict future outcomes. Examples from our review include forecasting future healthcare expenditures, predicting hospitalizations or emergency department visits, and assessing mortality risks.
2. Construct validity: This approach involved evaluating a tool’s ability to segment a population into distinct, meaningful groups. Validation was demonstrated by showing statistically significant differences in healthcare utilization patterns, demographic profiles, or clinical complexity across the generated segments.

## Figures and Tables

**Table 1:** Phases of the PICO process.

Element	Description
**Population**	Patients of all ages with chronic diseases
**Intervention**	Application of the population health management (PHM) technique
**Comparison**	Other available tools or no intervention
**Outcome**	Reduction in healthcare costs (e.g., fewer hospitalizations, better management)
**Application**	Applicable across various clinical settings and chronic patient populations

**Table 2:** Highlighted stratification tools and their breakdown.

Tool/Model	Method	Stratification Type	Peer Review	Owner	Needs Full EHR	# Segments
Bridges to Health [5]	Expert	Clinical	✘	✘	✘	8
COMPLEXedex [6]	Expert	Clinical, Lifestyle	✘	✔	✔	4
SSA—Senior Segmentation Algorithm	Expert	Clinical	✔	✔	✔	3
Delaware Clustering [10]	Expert	Clinical	✘	✘	✔	20
Lombardy Region Segmentation [11]	Guided Expert	Clinical, Demographic	✘	✘	✔	8
3M™ Clinical Risk Groups [12]	Guided Expert	Clinical, Demographic	✔	✔	✔	6–269
Medicare Segmentation [13]	Guided Expert	Clinical, Fragility, Demographic	✔	✘	✔	6
British Columbia Matrix	Guided Expert	Clinical, Demographic	✘	✘	✔	14
Singapore MOH Segmentation [14]	Expert	Clinical, Utilization	✔	✘	✔	6
North West London Segmentation	Data + Expert	Clinical, Demographic	✘	✘	✔	10
ACG—Adjusted Clinical Groups	Data + Expert	Clinical, Demographic	✔	✔	✔	92
Demand-Driven Model [15]	Data-Driven	Clinical, Functional	✔	✘	✘	5
LCA (Taiwan NHIS) [16]	Data-Driven	Clinical, Functional, Socio-demographic	✔	✘	✘	4
LCA (SIPA) [17]	Data-Driven	Clinical, Functional, Socio-demographic	✔	✘	✘	4
Utilization-Based Segmentation [18]	Data-Driven	Utilization	✘	✘	✔	8
Utilization/Demographic [19]	Data-Driven	Utilization, Demographic	✔	✘	✔	5
Davis et al. [20]	Data-Driven	Clinical	✘	✘	✔	7–53
Whitson et al. [21]	Data + Expert	Probabilistic	✘	✘	✔	6
Dorr et al. [22]	Data-Driven	Clinical, Probabilistic	✔	✘	✘	2
RiskER—Emilia-Romagna Region [23]	Data-Driven	Probabilistic	✘	✘	✔	4
CCSST [21]	Data-Driven	Clinical	✘	✘	✘	5–20

## Data Availability

The data analyzed in this systematic review consist of published articles and are available in the scientific databases searched, as specified in the Section 2 (PubMed, Cochrane, and Web of Science). The studies included in the analysis are cited in the References section.

## Supplementary Materials

The following supporting information can be downloaded at: https://www.mdpi.com/article/10.3390/medsci13030145/s1, PRISMA 2020 Main Checklist and the PRISMA Abstract Checklist. All references in these Supplementary Materials have been included in the reference list of the main text.
